# Neurofeedback for ADHD: Exploring the Role of Quantitative EEG and Brainwave Modulation

**DOI:** 10.1002/brb3.70714

**Published:** 2025-08-04

**Authors:** Rukiye Ölçüoğlu

**Affiliations:** ^1^ Faculty of Medicine, Department of Physiology Baskent University Ankara Turkey

**Keywords:** attention‐deficit hyperactivity disorder, brain modulation, neurofeedback, quantitative electroencephalography

## Abstract

**Purpose:**

Attention‐deficit/hyperactivity disorder (ADHD) is a complex and prevalent neurodevelopmental disorder characterized by inattention, hyperactivity, and impulsivity, often persisting into adulthood. This review aims to examine the neurobiological basis of ADHD, the role of quantitative electroencephalography (qEEG) in identifying biomarkers, and the clinical utility and challenges of neurofeedback (NFB) as a non‐pharmacological intervention.

**Method:**

A narrative review approach was employed to synthesize findings from recent neuroimaging and electrophysiological studies. Particular emphasis was placed on brain regions implicated in ADHD (e.g., prefrontal cortex, caudate nucleus, thalamus), EEG‐based biomarkers (notably the theta/beta ratio), and methodological factors influencing the efficacy of NFB protocols.

**Findings:**

Evidence suggests that NFB, grounded in operant conditioning, can modify dysfunctional brainwave patterns associated with ADHD. qEEG serves as a valuable tool for both identifying neurophysiological subtypes (e.g., cortical hypoarousal, hyperarousal, delayed maturation) and tailoring NFB protocols. However, heterogeneity in protocols, placebo effects (∼40% short‐term improvement), and variability in ADHD neurophysiology present significant challenges to standardisation and generalisability.

**Conclusion:**

While neurofeedback shows promise in reducing ADHD symptoms, its long‐term efficacy and comparability to pharmacological treatments remain inconclusive. Future large‐scale, well‐controlled trials are needed to establish robust, standardized protocols. Integrating NFB within a personalized, multimodal treatment framework may enhance clinical outcomes, particularly when tailored to EEG‐based ADHD subtypes.

## Graphical abstract

This review explores the efficacy of neurofeedback (NFB) in ADHD by analyzing key studies, EEG‐based subtypes, and protocol outcomes. Theta/beta and SMR training have been demonstrated to yield subtype‐specific benefits. Although there is evidence of acute improvements, including placebo effects, the long‐term efficacy of the treatment remains a subject of debate. Integration into multimodal treatment is recommended.

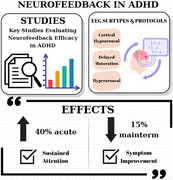

## 1 Introduction

Attention‐deficit/hyperactivity disorder (ADHD) is a prevalent neurodevelopmental disorder characterized by developmentally inappropriate inattention, hyperactivity, and impulsivity resulting in functional impairments across multiple domains (American Psychiatric Association 2013; Posner et al. 2020). Rather than being considered a discrete entity, ADHD is now recognized as a heterogeneous constellation of symptoms arising from diverse genetic, neurobiological, and environmental pathways (Thapar et al. 2022; Gallo and Posner 2016). This heterogeneity is evident in the varying responses to treatment, including to neurofeedback (NFB) interventions (Catalá‐López et al. 2017; Drechsler et al. 2020). ADHD often co‐occurs with other psychiatric disorders, and thus represents a significant burden for individuals, families, and society (Gallo and Posner 2016; Owens et al. 2020).

From a neurobiological perspective, ADHD is characterized by dysregulation across cortical–subcortical networks that govern executive function, attention, and impulse control (Faraone et al. 2015; Norman et al. 2025). The prevailing consensus in the field, as evidenced by the extant literature, is that structural and functional imaging consistently implicates dysfunction in the prefrontal cortex (PFC), particularly in the dorsolateral and anterior cingulate regions, in cases of impaired decision‐making and inhibitory control (Sridhar et al. 2017; Norman et al. 2025). Subcortically, there is a correlation between volume reductions in striatal nuclei (caudate, putamen) and thalamic‐prefrontal dysconnectivity, with symptom severity (Boedhoe et al. 2020; Parlatini et al. 2023). These alterations are exacerbated in comorbid conditions such as epilepsy, involving broader networks including the hippocampus and brainstem (Saute et al. 2014). The neurobiological heterogeneity of ADHD necessitates personalized interventions, as standardized approaches show inconsistent efficacy (Kiiski et al. 2020).

These structural and functional network abnormalities are closely related to dysregulations in monoaminergic (dopaminergic/noradrenergic) systems that regulate attention and behavioral control. ADHD involves dysregulation of monoaminergic neurotransmitter systems, particularly dopaminergic and noradrenergic pathways that modulate attention, motivation, and behavioral control (Del Campo et al. 2016). Evidence from neuroimaging and genetics confirms the presence of abnormalities in the striatal dopamine transporter, and adrenergic receptor gene polymorphisms (e.g., ADRA2A) are correlated with the severity of symptoms (Volkow et al. 2009; Cabana‐Domínguez et al. 2022; Yuan et al. 2021). Dopamine has been shown to play a critical regulatory role in the processing of reward, as well as in motor planning and the expression of novelty‐driven behavior, with these functions being facilitated through mesocorticolimbic circuits (Arnsten 2021). By contrast, norepinephrine has been found to optimize signal detection and task engagement via projections from the locus coeruleus to prefrontal regions. These neurotransmitter imbalances have been demonstrated to contribute to dysfunctional executive control networks, including fronto‐striato‐thalamic and fronto‐parieto‐cerebellar circuits. These networks exhibit reduced connectivity and activation during tasks requiring inhibition, working memory, and cognitive flexibility (Faraone et al. 2015; Norman et al. 2025).

The aetiology of executive dysfunction in ADHD is hypothesized to arise from distributed abnormalities across frontal‐striatal‐thalamic and fronto‐parieto‐cerebellar circuits, involving key nodes such as the anterior cingulate cortex and dorsal striatal structures (caudate, putamen) (González et al. 2021; Albajara Sáenz et al. 2019; Hoogma et al. 2017). Large‐scale consortium studies (ENIGMA‐ADHD) have confirmed consistent volume reductions in striatal structures across a sample of over 12,000 individuals (Boedhoe et al. 2020), while decreases in grey matter have been localized to superior frontal, parietal, and limbic regions (Yu et al. 2023). As posited by Parlatini et al. (2023), white matter dysconnectivity in fronto‐striato‐cerebellar tracts has been demonstrated to result in further disruption to executive network communication. Neuroimaging meta‐analyses converge on three key findings:

- Thalamocortical hyperconnectivity and frontoparietal hypoconnectivity at rest (Norman et al. 2025)
- Hypoactivation in fronto‐striatoparietal circuits during executive tasks (Rubia 2018; Norman et al. 2025)
- Striatal‐prefrontal dysconnectivity correlating with symptom severity (Norman et al. 2025).

These multimodal abnormalities extend to the amygdala and cerebellum, forming a neurobiological signature that maps onto clinical deficits in inhibition, working memory, and cognitive flexibility (Faraone et al. 2015; Norman et al. 2025).

These neuroimaging advances have enabled clinically applicable tools such as quantitative electroencephalography (qEEG), which directly links neural circuit abnormalities and real‐time brain dynamics. In contrast to the spatial precision afforded by fMRI, qEEG provides millisecond‐resolution monitoring of electrical oscillations during both rest and task states. Source‐localized high‐density electroencephalograms (EEGs) have been shown to map ADHD‐characteristic theta/beta imbalances to the executive network hubs of the fronto‐striatal and posterior parietal regions (McVoy et al. 2019; Mazaheri et al. 2014; Kiiski et al. 2020). This provides direct electrophysiological correlates of network dysfunction. The canonical ADHD qEEG profile of elevated theta (4‐8 Hz) and reduced beta (13–30 Hz) power, particularly during rest, robustly correlates with behavioral measures of inattention and impaired cognitive control (Lansbergen, Arns, et al. 2011; Arns et al. 2013; Kiiski et al. 2020).

While the default mode networks have been demonstrated to contribute to the pathophysiology of ADHD (Rubia 2018), qEEG most directly captures executive network dysregulation, making it particularly suitable for NFB targeting.

## 2 Review Methodology

This narrative review synthesizes evidence through critical analysis rather than systematic methodology, focusing on conceptual development and clinical implications in ADHD NFB. Although the formal PRISMA guidelines were not employed, the study selection prioritized the following:

- Seminal works establishing foundational NFB principles
- High‐impact clinical trials (RCTs with >30 participants)
- Recent meta‐analyses (2018–2023)
- Controversial studies highlight ongoing debates

Table 1 is designed to cover key studies reflecting protocol diversity. Randomized controlled trials (RCTs), studies with 30+ participants, and meta‐analyses from 2018 to 2023 were prioritized. As illustrated in Table 1, a comprehensive overview of key studies examining the efficacy of NFB in the treatment of ADHD is presented. Each entry comprises the study type, sample characteristics, outcome measures, observed effect sizes, and key findings. This enriched structure facilitates more transparent comparisons across various protocols and findings, thereby offering a more rigorous and methodologically sound assessment of clinical relevance.

**TABLE 1:** Key studies evaluating neurofeedback efficacy in ADHD.

Author (Year)	Study type	Sample and protocol	Outcome measure	Effect size	Key findings
Lubar and Shouse (1976)^a^	CS	1 child; SMR NFB	Attention	N/A	↑ Attention (clinical observation)
Monastra et al. (2005)	CT	*n* = 100 children; T/B NFB	Attention & executive function	*d* = 0.71 [0.42–1.00]	↑ Attention 40%; ↑ Executive Function 35%
Arns et al. (2009)	MA	10 studies (*n* = 409); T/B NFB	ADHD core symptoms	*d* = 0.63 [0.39–0.87]	Moderate symptom reduction (ES = 0.63)
Arns et al. (2014)	RCT	*n* = 41 children; SMR vs. T/B	Hyperactivity & attention	*d* = 0.58 [0.21–0.95]	SMR ↓ Hyperactivity; T/B ↑ Attention
Bink et al. (2016)	FU	*n* = 25 adolescents; SCP NFB	General ADHD Symptoms	*d* = 0.49 [0.12–0.86]	Sustained effects at 1 year
Catalá‐López et al. (2017)	Network MA	*n* = 1981; various NFB	Pharma vs. non‐pharma	OR = 1.14 [0.98–1.32]	Pharmacological superiority
Arnold et al. (2021)	RCT	*n* = 189 children; Sham‐controlled	ADHD symptoms	Placebo *d* = 0.40 [0.22–0.58]	40% acute improvement from placebo effects
Riesco‐Matías et al. (2021)	MA	17 studies (*n* = 1203); T/B NFB	Inattention	*d* = 0.55 [0.38–0.72]	Inattention reduction; medication superior
Ebrahim Rahmani et al. 2022)	SR & MA	23 studies (*n* = 1576)	General ADHD symptoms	*g* = 0.32 [0.21–0.43]	No clear advantage over other treatments
Louthrenoo et al. (2022)	MA	15 studies (*n* = 782); executive NFB	Executive functions	SMD = 0.45 [0.29–0.61]	Positive trend in executive functions
Garcia‐Pimenta et al. (2021)	Critical Review	*n* = 11 RCTs; multimodal NFB	Symptom remission	RR = 2.1 [1.6–2.7]	32%–47% remission rate
Lee et al. (2023)	MA	18 studies (*n* = 1142); T/B NFB	Attention	*g* = 0.61 [0.42–0.79]	Significant attention gains (*p* < 0.001)

Abbreviations: CS, case study; CT, controlled trial; *d* = Cohen's *d*; FU, follow‐up; *g*, Hedge's *g*; MA, meta‐analysis; OR, odds ratio; RCT, randomized controlled trials; RR, relative risk; SCP, slow cortical potential; SMD, standardized mean difference; SMR, sensorimotor rhythm; SR, systematic review; T/B, theta/beta.

^a^ Studies of historical significance are also included.

This methodological framework informs our critical examination of qEEG's role in identifying ADHD subtypes and guiding NFB protocols.

## 3 Quantitative EEG in ADHD: From Neurophysiological Markers to Therapeutic Applications

The capacity of qEEG to quantify real‐time neurophysiological deviations makes it particularly valuable for the identification of ADHD biomarkers and the guidance of therapeutic interventions. By analyzing oscillatory patterns (absolute/relative power, coherence), qEEG is capable of detecting state‐dependent neural dysregulation that frequently evades detection by structural imaging (Coben and Evans 2011; Lenartowicz and Loo 2014). This approach is founded on the hypothesis proposed by Berger a century ago, which posits that abnormal EEG patterns are indicative of psychopathology. The hypothesis that ADHD is associated with frontal cortical slowing is supported by eight decades of consistent evidence (Evans and Abarbanel 1999; Arns et al. 2013).

The utilization of Fourier or wavelet analysis facilitates the comparison of brainwave patterns, encompassing absolute and relative EEG power, coherence, and peak alpha frequency, with reference databases. This methodology allows researchers and clinicians to identify deviations in brain activity that may be clinically significant for ADHD (Coben and Evans 2011). The application of computer‐based techniques for the monitoring of EEG activity in humans has enabled the identification of profiles associated with specific patterns in a range of psychiatric and neurological disorders (Evans and Abarbanel 1999), aligning with modern psychiatry's biomarker identification goals using multimodal techniques (Kropotov 2009).

Recent research calls into question the reliability of the theta/beta ratio (TBR) as an ADHD biomarker (Simkin et al. 2014), particularly in light of the inconsistent results of the FDA‐approved NEBA system and the evidence suggesting that it is constrained in its development to childhood (Kiiski et al. 2020). Elevated theta/TBR is indicative of impaired task‐activation rather than global hypoarousal (Liechti et al. 2013; Arns et al. 2013).

Cluster analysis has identified several subtypes within the ADHD population, as defined by EEG, including a cortical hypoarousal subtype, a delayed maturation subtype, and a hyperarousal subtype (Holtmann et al. 2014; Clarke et al. 2001). The hypoarousal subtype is distinguished by elevated total power, augmented relative theta, and a high TBR, indicative of diminished cortical arousal. The delayed maturation subtype is characterized by increased slow‐wave activity and reduced fast‐wave activity, which suggests a postponement of central nervous system maturation. The hyperaroused subtype is distinguished by an excess of beta activity (Kropotov 2016b). These profiles correlate with fronto‐cortical slowing (González et al. 2021) and delta increase (Bashiri et al. 2018), though frequency boundaries remain complex (Güntekin and Başar 2016).

Table 2 translates these subtypes into clinical practice by aligning EEG profiles with evidence‐based NFB protocols, training durations, and target parameters. Specifically, TBR abnormalities in hypoarousal directly inform theta/beta protocol selection, while delayed maturation guides sensorimotor rhythm (SMR) training.

**TABLE 2:** Suggested neurofeedback protocols by EEG subtype.

ADHD EEG Subtype	Protocol	Training duration	EEG features	Expected outcomes	Key evidence
**Cortical hypoarousal**	Theta/Beta NFB	20–40 sessions	↑Theta, ↓Beta, ↑TBR	↑Sustained attention; ↓TBR	Monastra et al. (2005); Arns et al. (2014)
**Delayed maturation**	SMR NFB	30–40 sessions	↑Slow‐wave, ↓Fast‐wave	↑SMR power; ↓Hyperactivity	Lubar and Shouse (1976); Gevensleben et al. (2014)
**Hyperarousal**	SCP NFB	30+ sessions	↑Beta, ↓SCP amplitude	↑Self‐regulation; Symptom stability	Bink et al. (2016); Garcia‐Pimenta et al. (2021)

Abbreviations: SCP, slow cortical potential; SMR, sensorimotor rhythm.

**FIGURE 1:**
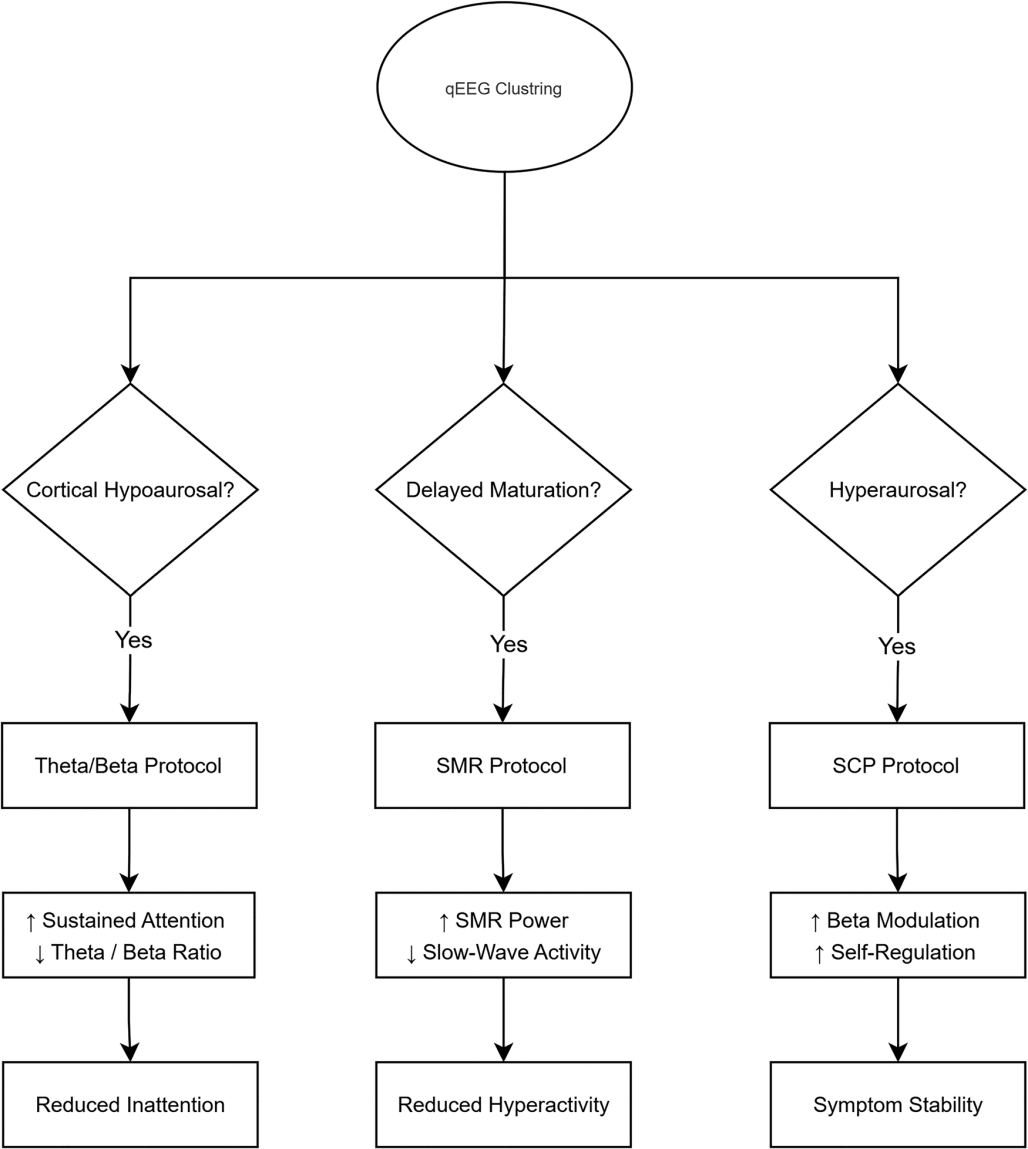
Evidence‐based neurofeedback protocol selection flowchart based on qEEG subtypes. (Decision pathways reflect clinical recommendations from Table 2).

The following flowchart (Figure 1) illustrates how qEEG findings guide the selection of NFB protocols for ADHD subtypes. First, the qEEG clustering method assigns patients to one of three EEG‐based subtypes (cortical hypoarousal, delayed maturation, or hyperarousal). Each of these subtypes is associated with a specific protocol, namely theta/beta, SMR, or slow cortical potential (SCP), and the primary neurophysiological and behavioral effects are outlined below.

In conclusion, the application of subtyping or clustering approaches to qEEG deviations facilitates a more nuanced comprehension of ADHD as a disorder characterized by substantial heterogeneity (Clarke et al. 2011). Nevertheless, the longitudinal stability of EEG frequency band changes associated with ADHD from childhood to adulthood has been the subject of recent debate (Liechti et al. 2013; Poil et al. 2014). Given the capacity of qEEG to delineate neurophysiological abnormalities, it is imperative to explore how these findings can be applied therapeutically. The following section will examine the mechanisms and applications of NFB, with a particular focus on its potential for treating ADHD.

## 4 NFB as a Therapeutic Approach: Mechanisms and Applications

NFB employs operant conditioning principles to train self‐regulation of brain activity. Real‐time EEG parameters are presented to the subject via visual and auditory signals, thereby enabling the subject to voluntarily modulate cortical oscillations that are linked to cognitive functions (Sitaram et al. 2017; Davelaar 2022). This process utilizes the phenomenon of neuroplasticity to normalize dysfunctional networks in ADHD, with fMRI studies confirming protocol‐specific changes in prefrontal‐striatal circuits post‐training (Ros et al. 2010; Meir‐Hasson et al. 2016). EEG signals are processed in real‐time to extract target parameters (e.g., TBR). Feedback is dynamically adjusted to reinforce desired brain states, thereby creating a closed‐loop system in which the brain learns to self‐correct dysregulated patterns. For example, reducing theta amplitude while increasing beta enhances attentional control during game‐based tasks (Kropotov 2016b).

The efficacy of NFB is predicated on three principles that are underpinned by empirical evidence (Kropotov 2009, 2016b):

1. EEG‐Behavior Coupling: Specific oscillations have been shown to map to cognitive functions (e.g., theta suppression → attention) (Arns et al. 2013).
2. Dopaminergic Reinforcement: Successful self‐modulation of the brain triggers reward pathways (Volkow et al. 2009)
3. Neuroplastic Consolidation: Repeated training has been demonstrated to induce structural reorganization in thalamocortical networks (Gevensleben et al. 2014)

NFB is a process that leverages the brain's capacity for neuroplasticity, defined as the ability to reorganize neural connections through experience. As with music or meditation training, repeated NFB sessions induce enduring structural/functional changes by reinforcing specific oscillation patterns (Ros et al. 2010; Davelaar 2018). This operant conditioning process gradually affects a rewiring of dysfunctional circuits:

- Implicit learning (conditioning and repair): Automatic modulation of brain activity has been demonstrated to correct neural deficits without the need for conscious effort. An example of this phenomenon is provided by Gevensleben et al. (2014), theta reduction occurs during SMR training.
- Explicit learning (skill acquisition): The deliberate cultivation of self‐regulation strategies is paramount for the generalization of these skills to daily life, as evidenced by their application in the maintenance of attention (Hasslinger 2022). This process is of critical importance for the long‐term maintenance of these skills.

Dopamine release has been demonstrated to trigger neuroplasticity in thalamocortical circuits, thereby establishing the foundation for irreversible alterations. Dopamine release during successful trials has been shown to reinforce target patterns (Volkow et al. 2009), while the heightened neuroplasticity observed in childhood makes early intervention critical for the development of executive function (Sonuga‐Barke and Halperin 2010; Norman et al. 2025).

Traditional functional neuroimaging techniques are employed to identify correlations between brain activity and behavior. In contrast, NFB involves the direct manipulation of neural activity as an independent variable. By modulating the amplitudes of EEG and neuronal synchronization, NFB facilitates targeted self‐regulation of distributed functional networks implicated in psychiatric disorders, thus offering network‐level precision that is superior to that of anatomically nonspecific pharmacotherapy (Sitaram et al. 2017).

Despite extensive clinical validation, theoretical models explaining how individuals learn voluntary brain signal control remain underdeveloped. Schmorrow et al. (2020) first proposed a multi‐stage NFB learning theory comprising three phases:

1. Striatal learning: The initial reward‐based strategy selection was reinforced by dopamine release.
2. Thalamic consolidation: The process of sleep‐dependent restructuring of thalamocortical synapses via inhibitory plasticity.
3. Interoceptive homeostasis: The process of self‐calibration, facilitated by subjective awareness of internal states, has been demonstrated to be an effective method of achieving real‐world symptom stabilization.

Davelaar (2022) categorizes learning mechanisms into three frameworks: The first element is high‐level theories that identify universal principles across protocols. Second, cognitive theories focus on the impact of specific processes within a particular domain. Third, the examination of the contributions of discrete brain regions according to neural theories. Davelaar's (2022) cognitive/neural theory classification is consistent with Schmorrow's multi‐stage learning model and incorporates the universal principles of NFB. To encompass all three classes, a multi‐stage NFB learning theory (Schmorrow et al. 2020) has been developed with the objective of guiding efforts to understand the complexities of the NFB paradigm.

It is assumed that these phases commence in the order; however, it is not assumed that one phase will be completed before the next begins. Conversely, the three phases constitute an integrated, self‐regulating system with the capacity to modify the adjustment points of brain dynamics and maintain these new adjustment points over a range of timescales (Davelaar 2022; Schmorrow et al. 2018, 2020).

Although the mechanism specificity of NFB is well‐established, further research is required to determine its long‐term efficacy in comparison to stimulant medications, in order to facilitate its clinical translation. Section 5 of this text employs a comparative meta‐analysis and durability study (>12 months) to evaluate the aforementioned claims.

## 5 Long‐Term Efficacy of NFB in ADHD

Prior to conducting a comprehensive review of the extant literature on the long‐term efficacy of NFB in ADHD, a synthesis of comparative findings from foundational studies assessing both immediate and follow‐up outcomes has been conducted. Table 3 presents a comparison of the acute effects observed post‐session with the longer‐term clinical outcomes at ≥6 months. This framework provides a contextual framework for debates on NFB durability, demonstrating that SCP protocols demonstrate symptom stability at one‐year follow‐up (Bink et al. 2016), particularly for hyperarousal subtypes.

**TABLE 3:** Acute and long‐term effects of neurofeedback protocols.

Study (Year)	Protocol	Symptom target	Acute effects (post‐session)	Long‐term effects	Follow‐up duration
Monastra et al. (2005)	Theta/Beta	Sustained Attention	↑ Sustained attention immediately	N/A	None reported
Arns et al. (2014)	SMR	Impulsivity	↓ Motor impulsivity; ↑ SMR power	↑ SMR power maintained	6 months
Bink et al. (2016)	SCP	Global ADHD symptoms	N/A	↓ ADHD symptom scores	12 months
Arnold et al. (2021)	Sham NFB	Placebo Effects	40% symptom improvement	15% maintenance (6 mo)	6 months
Lee et al. (2023)	Theta/Beta	Attention	Significant gains	*d* = 0.59 maintenance	6 months

Abbreviations: SCP, slow cortical potential; SMR, sensorimotor rhythm.

Although the placebo effect is known to diminish over time (from 40% to 15% at six months), this suggests that NFB relies on persistent neural changes. However, the superiority of pharmacological treatments in key symptom domains should not be overlooked (Arnold et al. 2021; Riesco‐Matías et al. 2021). Recent meta‐analyses (Lee et al. 2023; Arnold et al. 2021) have provided a synthesis of the extant evidence from 32 RCTs. These meta‐analyses have demonstrated that theta/beta protocols have been found to maintain attention improvements at the 6‐month mark (*d* = 0.59), while SMR training has been found to sustain hyperactivity reduction at the 12‐month mark (*d* = 0.55). Furthermore, SCP has been found to achieve 32%–47% symptom remission (Garcia‐Pimenta et al. 2021). Notwithstanding these findings, however, sham‐controlled trials (Arnold et al. 2021; *n* = 189) reveal that 40% of acute improvements can be attributed to placebo effects, diminishing to 15% at 6‐month follow‐up (Arnold et al. 2021). Pharmacological interventions have been demonstrated to demonstrate superiority in core symptom domains (Riesco‐Matías et al. 2021), though NFB has been shown to offer complementary benefits in terms of executive function.

Early meta‐analyses (Arns et al. 2009) suggested that NFB outcomes were comparable to those of stimulants with regard to core ADHD symptoms. Lee et al. (2023) provided confirmation of the hypothesis that theta/beta NFB enhances attention in children, while Dobrakowski and Łebecka (2020) demonstrated that personalized NFB yields working memory improvements that persist for a minimum of one year. Louthrenoo et al. (2022) observed positive response inhibition trends, suggesting that session frequency and intensity have a significant impact on outcomes. However, Bink et al. (2016) found no significant NFB advantage over alternatives, and Ebrahim Rahmani et al. (2022) reported no superior efficacy versus other treatments. Riesco‐Matías et al. (2021) concluded that while NFB mitigates inattention, stimulants remain superior for the management of the full symptom spectrum. Garcia‐Pimenta et al. (2021) proposed a research agenda that explored the potential synergies between NFB and behavioral/pharmacological therapies. As Geladé et al. (2017) and Drechsler et al. (2020) have demonstrated, ADHD is characterized by a high degree of intrinsic complexity. However, the efficacy of stimulants in enhancing attention and inhibition has been well‐documented. As Geladé et al. (2018) emphasized, there is a necessity for further research to be conducted into the long‐term benefits of NFB, with a particular focus on its impact on working memory. The question of whether pharmacotherapy is superior to other treatment options in key domains remains a critical consideration for healthcare professionals.

Systematic reviews (Catalá‐López et al. 2017) identify NFB as a promising yet under‐researched field. Sibley et al. (2014) provide a contextual framework for understanding ADHD treatment, highlighting the potential of behavior therapy to address impairments more comprehensively than medications in certain cases. However, they also find no evidence to support the efficacy of NFB for adolescents. The necessity for standardization remains paramount, as evidenced by Geladé et al. (2018), who emphasized the impact of disparate evaluation criteria on outcome variability. The presence of patient heterogeneity and the inconsistency of EEG findings present a significant challenge to the development of conclusive evidence for NFB. Atypical resting‐state EEG patterns in ADHD further complicate the development of a universal protocol (Ogrim et al. 2014; Kanazawa 2014; Mazaheri et al. 2014). The rigorous validation of neuroimaging findings remains imperative. Concerning the methodology, inconsistencies in qEEG filter cut‐offs have been observed, which consequently affect the interpretation of frequency bands. The American Academy of Neurology and the American Psychiatric Association advocate the utilization of qEEG as an adjunct to standard EEG (Nuwer 1997). Addressing these challenges is imperative for the broader acceptance of clinical NFB.

Despite the existence of favorable long‐term outcomes in certain studies, debates regarding the efficacy of NFB persist, largely due to methodological inconsistencies, patient variability, and generalizability issues. It is imperative that these issues are addressed in order to establish NFB as a reliable treatment for ADHD.

## 6 Methodological Challenges in NFB Research and Practice

The use of NFB therapy has gained considerable attention due to the hypothesis that neuropsychological disorders originate from nervous system dysfunction. Consequently, research has been conducted on a range of conditions, including ADHD, epilepsy, autism spectrum disorder, depression, and anxiety (Hughes and John 1999; Tan et al. 2009; Holtmann et al. 2011). Furthermore, NFB has been employed in healthy populations with the objective of enhancing cognitive functions, including memory, attention, and performance optimization in a range of fields (Gruzelier 2014a, 2014b; Marzbani et al. 2016).

Despite the promising results observed in many NFB studies, a number of methodological challenges persist, including the use of small sample sizes, limited training sessions, lack of blinding, and significant variability across ADHD subtypes (Micoulaud‐Franchi et al. 2015; Omejc et al. 2019; Begemann et al. 2016; Niv 2013). The lack of consistency in the descriptions of protocols, the difficulties in controlling for placebo effects, and the absence of standardized parameters for key aspects of NFB, such as electrode placement and session duration, have hindered the ability to definitively establish its efficacy. A further crucial factor to be taken into account is the possibility of placebo effects. As with other forms of behavioral therapy, NFB requires the use of specialized equipment and regular clinician interaction, which may contribute to improvements that are not directly related to the regulation of the brain. Studies utilizing a sham NFB (where feedback is random or pre‐recorded) have demonstrated that patients can exhibit comparable improvements to those undergoing genuine NFB (Arnold et al. 2021; Liechti et al. 2012; Lansbergen, et al.^2011^; Schönenberg et al. 2017). Specifically, Arnold et al. (2021) found that 40% of acute symptom improvement in NFB groups was attributable to placebo effects, though this decreased to 15% at 6‐month follow‐up. This indicates that psychosocial factors, such as patient expectations and interaction with clinicians, may have a substantial influence on the observed outcomes (Thibault et al. 2018; Thibault and Raz 2016). Moreover, the extrapolation of NFB‐induced behavioral changes to everyday life remains challenging due to heterogeneous patient responses, as many studies lack the double‐blind RCTs that are essential for the exclusion of placebo effects (Arnold et al. 2021; Thibault et al. 2018). Furthermore, long‐term outcomes remain underexplored due to a paucity of follow‐up assessments (Omejc et al. 2019; Marzbani et al. 2016).

This is particularly evident in the context of ADHD research, where substantial neurophysiological heterogeneity complicates the standardization of research protocols. As Kiiski et al. (2020) have noted, distinct EEG profiles—that is, cortical hypoarousal versus hyperarousal—respond differentially to similar protocols, thus contributing to inconsistent outcomes across studies.

Nevertheless, NFB shows promise, particularly when integrated into a comprehensive, multimodal treatment framework for ADHD. The final section will address the optimal incorporation of NFB into such a framework. It is imperative that these methodological limitations are addressed prior to the translation of NFB findings into clinical practice.

## 7 Discussion: Bridging the Gap Between NFB Promise and Clinical Reality

Although NFB has demonstrated considerable potential as a treatment for ADHD, there are still significant challenges in translating the findings from experimental studies to clinical practice. The rationale for utilizing NFB to treat ADHD is based on initial observations that children with ADHD demonstrated elevated amplitudes of low‐frequency EEG waves (e.g., delta and theta bands) in comparison to their typically developing counterparts (Poil et al. 2014; Dustman et al. 1999). Although pharmacotherapy can frequently diminish these elevated amplitudes, some patients experience inadequate symptom control or intolerable side effects. Furthermore, pharmacological interventions may not fully address all areas of impairment associated with ADHD (Ogrim et al. 2014; Janssen et al. 2016; Ghuman and Ghuman 2013). Consequently, NFB has emerged as a potential alternative or complementary treatment for these patients.

Since the initial reports of NFB's efficacy in treating ADHD (J. F. Lubar and Shouse 1976), a substantial body of research has explored the effects of various NFB protocols, focusing on core ADHD symptoms such as inattention, impulsivity, and hyperactivity. The majority of these studies employ protocols that target SMR, beta rhythm, or SCP training (Scott et al. 2005; J. O. Lubar and Lubar 1984; Logemann et al. 2010; Vernon et al. 2003; Monastra et al. 2005). However, ADHD is a heterogeneous disorder, and only approximately 30% of patients display an elevated TBR. This emphasizes the necessity for bespoke NFB protocols, as conventional NFB protocols may prove ineffective for a proportion of patients. While promising, personalized approaches still require validation through large‐scale RCTs incorporating qEEG subtyping (Kiiski et al. 2020; Garcia‐Pimenta et al. 2021).

A persistent challenge in NFB research is the lack of clarity surrounding the precise mechanisms through which NFB exerts its effects. One hypothesis is that patients may engage in compensatory mechanisms as a means of addressing the neural dysfunction associated with ADHD, rather than directly addressing the underlying neural dysfunction. For example, children who are trained to enhance frontal beta activity may learn to voluntarily focus their attention, thereby strengthening the neural networks that are involved in attention regulation (Nolan and Carr 2013). Notwithstanding the prevailing uncertainty with regard to the precise mechanisms involved, the combination of NFB with pharmacotherapy and behavioral interventions may prove efficacious in addressing the heterogeneous nature of ADHD (Garcia‐Pimenta et al. 2021). Recent critical reviews emphasize that NFB achieves optimal outcomes when integrated into personalized multimodal frameworks combining NFB with pharmacotherapy and behavioral interventions (Garcia‐Pimenta et al. 2021).

Moreover, the considerable variability in NFB protocols, including frequency targets, electrode placement, and feedback mechanisms, represents a significant challenge. The choice of hardware and software also has an impact on the results. Any modification to these elements could impact the efficacy of the treatment, underscoring the importance of standardizing these parameters to ensure more consistent and reliable outcomes (Landes et al. 2017).

Despite the current limitations of NFB methodology, its potential merits should not be disregarded. To achieve this potential, NFB must be incorporated into a more comprehensive ADHD treatment framework. By combining NFB with other therapeutic modalities and leveraging advancements in neuroimaging, it is possible to develop more personalized and effective interventions for ADHD.

## 8 Future Directions: Integrating NFB Into Multimodal ADHD Treatment Approaches

The future of NFB as a treatment for ADHD is contingent upon its integration into a comprehensive, multimodal framework that encompasses pharmacological and behavioral therapies. Given the considerable heterogeneity of ADHD and the variability in individual responses to treatment, a one‐size‐fits‐all approach is unlikely to prove successful. Instead, NFB is most effective when tailored to the patient's neurophysiological profile and integrated with other therapeutic modalities.

As with pharmacological interventions, NFB should only be administered following a comprehensive diagnostic evaluation and under the supervision of a certified specialist. It is imperative to approach NFB with caution, as improper modulation of EEG rhythms has the potential to result in dysregulation and, subsequently, an exacerbation of symptoms (Kropotov 2016a, 2016b). The NFB process entails the acquisition of skills whereby patients learn to regulate their brain activity in a gradual manner. It is not uncommon for patients to initially experience difficulty in associating their emotional or cognitive states with the EEG feedback displayed during sessions. However, with practice, patients become proficient in maintaining the desired brain state, both during sessions and in their everyday environments, such as at home or school.

NFB treatment typically necessitates 20–40 h of intensive training, which requires considerable input from both the clinician and the patient. Motivation is a critical factor in the efficacy of NFB, as the quality of skill acquisition is largely contingent on the patient's engagement in the process.

Although placebo effects are an inherent aspect of NFB treatment, it is also crucial to acknowledge the potential role of specific neural mechanisms in the manifestation of these placebo‐like responses. It is imperative that future research places a premium on the implementation of double‐blind designs, complemented by active sham controls. This methodological emphasis is particularly salient in the context of NFB, where the objective is to discern specific NFB effects from the potential contributions of placebo responses. This necessity is underscored by the findings of Arnold et al. (2021), which revealed that a significant proportion (40%) of acute improvements are nonspecific, thereby highlighting the need for rigorous research methodologies to ensure the validity of observed outcomes. In certain instances, it may prove challenging to differentiate between the effects of NFB and those of a placebo in controlled trials or in evaluations conducted by blinded raters (Sonuga‐Barke et al. 2013).

Although uncontrolled studies have demonstrated promising outcomes, recent meta‐analyses have indicated that more robust evidence is required before NFB can be fully endorsed as a standalone treatment for ADHD. Specifically, qEEG‐guided protocols matched to ADHD subtypes (e.g., SCP for hyperarousal) show particular promise for optimizing outcomes (Bink et al. 2016; Garcia‐Pimenta et al. 2021). However, the inability to differentiate its effects from placebo in rigorous trials (Grus 2003) suggests that NFB should be employed as part of a multimodal approach to ADHD treatment, complementing pharmacological and behavioral therapies to provide a comprehensive solution for managing ADHD symptoms. Combining stimulant medication with targeted NFB protocols may be especially beneficial for cases that are treatment‐resistant (Catalá‐López et al. 2017).

## Author Contributions

**Rukiye Ölçüoğlu**: conceptualization, methodology, writing–original draft, writing–review and editing.

## Ethics Statement

The authors have nothing to report.

## Conflicts of Interest

The author declares no conflicts of interest.

## Peer Review

The peer review history for this article is available at https://publons.com/publon/10.1002/brb3.70714

## Data Availability

Data sharing is not applicable to this article as no new data were created or analyzed in this study.
